# The Ethiopian emergency medical team: its formation, progress, response experience and opportunities

**DOI:** 10.3389/fpubh.2025.1542303

**Published:** 2025-04-30

**Authors:** Boniface Oyugi, Nahom Tadelle, Alegnta Gebreyesus, Martinez-Monterrey Lazaro, Jerry-Jonas Mbasha, Pryanka Relan, Thierno Balde, Flavio Salio, Melkamu Abtea, Neima Zeynu, Joseph Okeibunor

**Affiliations:** ^1^World Health Organization, Emergency Medical Teams Initiative, Geneva, Switzerland; ^2^Centre for Health Services Studies (CHSS), University of Kent, Canterbury, United Kingdom; ^3^Ethiopian Emergency Medical Team, Ethiopian Public Health Institute, Addis Ababa, Ethiopia; ^4^World Health Organization, Ethiopia Country Office, Addis Ababa, Ethiopia; ^5^World Health Organization, Regional Office for Africa, Emergency Preparedness and Response Programme, Brazzaville, Democratic Republic of Congo; ^6^World Health Organization, Emergency Preparedness and Response Programme, Regional Hub for West Africa, Dakar, Senegal; ^7^Ethiopian Public Health Institute, Public Health Emergency Management (PHEM), Addis Ababa, Ethiopia

**Keywords:** Ethiopia, disaster management, response, emergency medical teams (EMTs), WHO African Region, public health emergencies

## Abstract

**Background:**

Ethiopia faces climate shocks, conflict, food insecurity, and limited livelihoods, creating urgent humanitarian needs and a critical demand for emergency medical response. A well-coordinated national emergency medical team (N-EMT) is essential to address these crises effectively.

**Objective:**

This study documents the progress of the country's emergency response mechanisms and N-EMT development, highlights lessons learned for other countries implementing emergency medical teams (EMTs) and concludes with recommendations for improvement.

**Methods:**

This study employed a holistic single-case study design, integrating mixed methods approaches to explore the introduction and establishment of N-EMT in the Federal Republic of Ethiopia. It examined the national disaster response context, highlighting key challenges, enabling factors, and emergent opportunities. Data were collected through key informant interviews (KIIs) and an in-depth review of relevant policy and operational documents. The study utilizes the guiding framework for implementing N-EMTs in addition to thematically documenting lessons learnt.

**Results:**

Launched in August 2018, the Ethiopian Disaster Medical Assistance Team (DMAT) initiative aimed to enhance Ethiopia's response to rising emergencies in peripheral regions by establishing a structured framework with trained professionals. This led to the creation of N-EMTs and a strategic implementation roadmap, supported by a core team executing a comprehensive joint work plan. Collaborating with partners and utilizing existing government systems ensured resource management and access to essential supplies. Strong backing from the Ministry of Health (MoH) and high-level government offices was vital for integration and sustainability, emphasizing political will's role in advancing health frameworks. The N-EMT expanded to address mass gatherings, conflicts, and malnutrition, enhancing capabilities and participating in regional health diplomacy. Collaborations with United Kingdom Medical Emergency Team (UK-MED) and Polish Center for International Aid (Polskie Centrum Pomocy Miedzynarodowej) (PCPM) refined verification, human resource (HR) management, and logistics, supported by innovative funding. Ethiopia's N-EMT implementation score reached 69 out of 96, indicating substantial progress toward full operationalization. Of the total implementation activities, 27 were fully completed, 15 were partially achieved or ongoing, and 6 had yet to commence. Key lessons learned emphasized the importance of streamlined resource management, the establishment of advance teams, robust preparedness measures, coordinated response mechanisms, and the provision of psychological support following deployments.

**Conclusion:**

Ethiopia has made strong and measurable progress in developing its N-EMT, establishing a foundational framework, mobilizing trained personnel, and expanding its scope to address a variety of emergencies. However, to reach full WHO classification, specific gaps remain—particularly in institutionalizing coordination structures, formalizing deployment protocols, and strengthening logistics and human resource systems. This experience highlights the importance of embedding N-EMTs within the national health and emergency response systems, backed by sustained political commitment, strategic partnerships, and dedicated investment in capacity building and preparedness infrastructure.

## 1 Background

**Figure 1:**
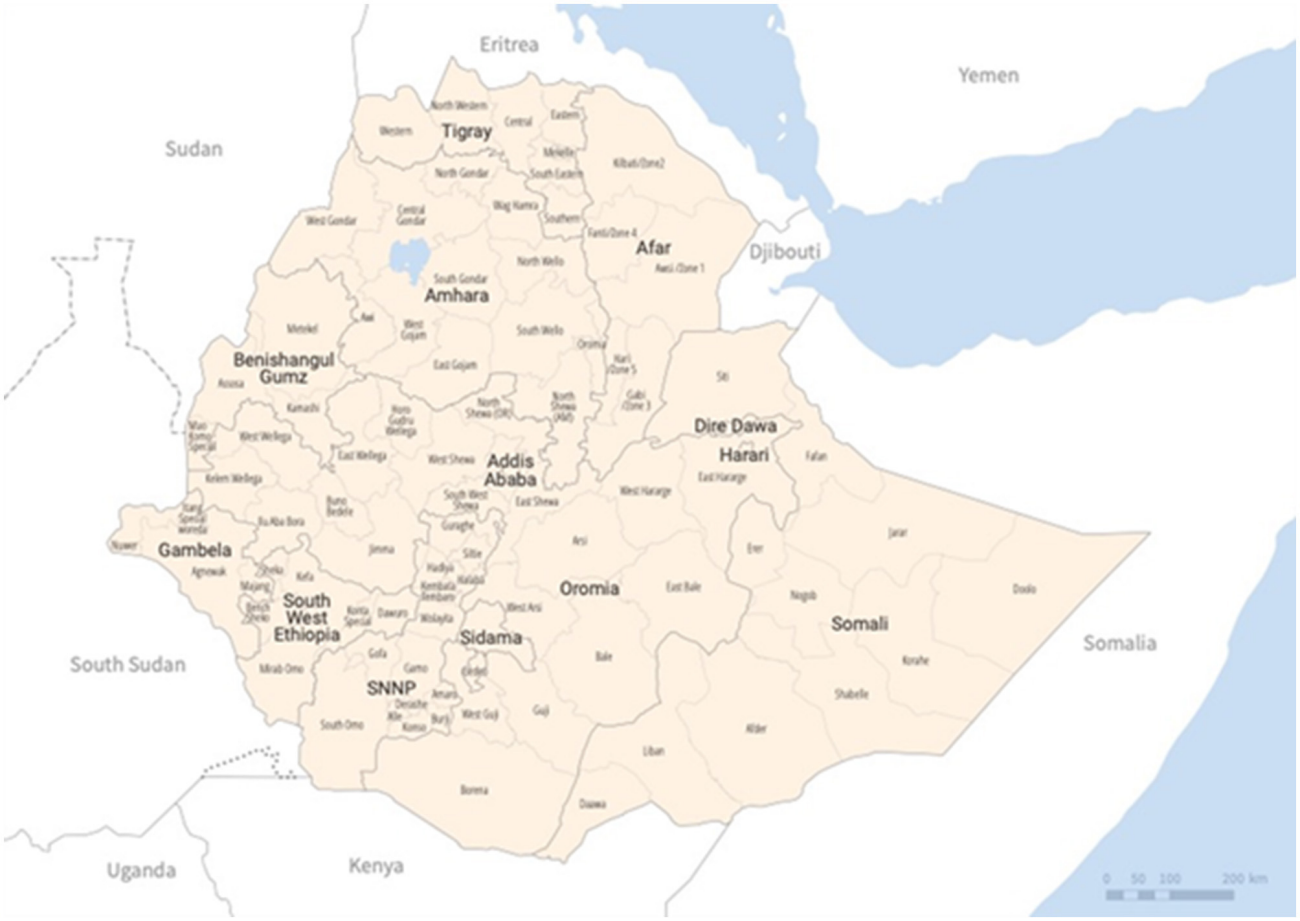
Map of Ethiopia [source: World Health Organization (WHO)]. Disclaimer: The designations employed and the presentation of the material in this publication do not imply the expression of any opinion whatsoever on the part of WHO concerning the legal status of any country, territory, city or, area or of its authorities, or concerning the delimitation of its frontiers or boundaries. Dotted and dashed lines on maps represent approximate border lines for which there may not yet be full agreement.

The Federal Democratic Republic of Ethiopia is situated in the Horn of Africa. It is bordered by Sudan to the northwest, South Sudan to the west, Somalia to the east and southeast, Eritrea to the north, Djibouti to the northeast, and Kenya to the south (Figure 1) (1) and is located between the Tropic of Cancer and the Equator, within the latitudes 30°N and 150°N, and longitudes 330°E and 480°E. The country covers an area of 1.1 million km^2^, with water bodies occupying 7,444 km^2^ (2). As of 2023, the estimated population is 126.5 million (about 1.57% of the world population) (3). Ethiopia experiences rapid population growth at a rate of 2.6%, and has a young age structure (2). The median age of the Ethiopian population is 18.8 years, and only 22.1% of the population lives in urban areas (3). The life expectancy at birth is 65 years, and the total fertility rate is 4.2 births per woman (4), corresponding to a crude birth rate of 32 per 1,000 and a crude death rate of 6.3 per 1,000 population (5). The average household size is 4.7 persons, with urban households slightly smaller than rural households (4.1 persons vs. 5.0 persons) (6). Most Ethiopian households (78%) are headed by men (6). Children under 15 years of age and individuals in the 15–65 age group make up 47% and 49% of the population, respectively, while only 4% of the population is over 65 years old (2). The sex ratio between males and females is almost equal, and women of reproductive age make up about 23% of the population (2).

By 2023, the country consisted of 12 National Regional States (NRS) and two city administrative councils. The 12 NRS are Afar, Amhara, Benishangul-Gumuz, Central Ethiopia, Gambela, Harari, Oromia, Sidama, Somali, South Ethiopia, Southwest, and Tigray. The city administrative councils are Addis Ababa and Dire Dawa. These regions are further divided into 68 zones and 770 districts (woredas), as shown in Figure 1.

According to the Human Development Index (HDI) for 2022, Ethiopia has a low rating of 0.492, placing it in the category of low human development, ranking 176 out of 193 countries and territories (7). The country has seen a positive change of 72% in HDI value between 2000 and 2022, with the rating increasing from 0.286 to 0.492 (7). Ethiopia faces a triple burden of diseases, including communicable and non-communicable diseases (NCDs), mental health issues, and injuries affecting all age groups, with a higher burden among children and women of reproductive age (5). Over the past two decades, Ethiopia has significantly improved women's and children's health, with maternal mortality ratios decreasing from 953 to 267 per 100,000 live births and the under-5 mortality rate dropping from 140 to 46 per 1,000 live births (8, 9).

The percentage of individuals covered by primary health services increased from 50.7% in 2000 to over 90% in 2019 (2). However, as of 2021, the universal health coverage (UHC) service coverage index is only 43% (2). In recent years, Ethiopia's out-of-pocket (OOP) health expenditure has decreased, reaching 33.09% in 2020 (10). In Ethiopia, health services are primarily delivered through a three-tier public system: primary healthcare units, which include district hospitals, health centers, and health posts staffed by community health workers (CHWs); secondary care, provided by general hospitals; and tertiary care, offered at comprehensive and referral hospitals (2). Over the past decade, the number of hospitals, health centers, and health posts providing services has significantly increased (2, 11). Ethiopia had 434 hospitals, 3,890 health centers, and 18,090 health posts in 2020, leading to improved access to health services for the people (12). The public health facilities employed 300,000 healthcare workers, with 210,000 (70%) being health professionals and the remaining 90,000 (30%) serving in administrative/support roles (13). The health professional to population ratio was one physician for every 8,000 people, one nurse for every 1,500 people, and one midwife for every 5,000 people (13). Additionally, over 45,000 health extension workers provided community health services in more than 18,000 health posts (13). The country has implemented healthcare financing reforms to raise sufficient financial resources, including exempted services, fee waiver systems, revenue retention and utilization, outsourcing services, establishing private wings, and adopting a community-based health insurance system (11).

Ethiopia is a diverse and populous country that faces several challenges, including climatic shocks, persistent conflict, food insecurity, and reduced access to livelihoods, leading to ongoing humanitarian needs. The emergency medical response has played a critical role in addressing the country's health crises. Between 2020 and 2022, five consecutive seasons of poor rainfall caused severe drought, exacerbating food and water shortages and impacting livelihoods and livestock. Furthermore, heavy rainfall in late 2023, associated with the El Niño phenomenon, led to flooding, damaging infrastructure, displacing households, and increasing the risk of waterborne diseases (14). The country's health system experiences recurring outbreaks of diseases such as measles, malaria, yellow fever, and cholera, further strained by the global COVID-19 pandemic. Intercommunal conflicts since 2018 and the conflict in northern Ethiopia have resulted in widespread displacement and hindered access to humanitarian aid, with many health facilities being damaged. The 2024 Ethiopia Humanitarian Response Plan (HRP) aims to raise $3.24 billion to assist 15.5 million people, including 4 million internally displaced persons (IDPs). The plan emphasizes the urgent need for assistance, especially for those affected by the El Niño-induced drought and ongoing conflicts (14).

The emergency medical response has been crucial in addressing these crises for instance through the provision of immediate medical care, nutritional support, and water purification. Rapid response units also helped flood-affected areas in 2023. Mobile health clinics have been vital in conflict-affected regions, delivering essential health services to internally displaced persons. However, the emergency medical response in the country requires better coordination compared to other public health response pillars. This lack of coordination leads to delays in mobilizing and deploying professionals. While some improvements have been made in emergency governance, there are still significant gaps that need to be addressed. These include the need for an organized and updated professional roster, operational standards, and adequate pre-positioning of medical equipment and supplies. It's important to learn from previous recovery phases to improve future readiness. Despite these challenges, the multi-hazard approach remains valuable and versatile and can be used in various scenarios such as mass gatherings, conflicts, and disease outbreaks.

This study documents the progress of the country's emergency response mechanisms and the development of the national EMT (N-EMT). It highlights the lessons learned from implementing the N-EMT, which can be used by other teams establishing and implementing their N-EMTs. The study concludes by reflecting on the results and proposing recommendations to improve the implementation of N-EMTs.

## 2 Methods

This study was conducted using a single case (holistic) study design, as defined by Yin (15), which investigates a real-world phenomenon within its natural context using one bounded case to explore complex systems in depth. This approach was well-suited for examining Ethiopia's development of a N-EMT, a unique and context-specific process. Ethiopia's experience was considered exceptional, revelatory, and representative—offering a comprehensive example of EMT establishment in a low-resource setting. Notably, Ethiopia was the first country in the WHO African Region to establish a functional N-EMT.

The case study design enabled a rich, longitudinal analysis by capturing the historical context, key milestones, and lessons learned during the country's health emergency response evolution. The parameters of the case included the historical background of Ethiopia's Health Disaster Response, the events leading to EMT establishment, the activities and deployments undertaken, and the lessons learned from these experiences. A key strength of this design was its ability to integrate multiple sources of evidence, thereby enhancing the study's overall validity and reliability. The use of a mixed methods approach—combining retrospective data/information from document reviews, and KIIs—enabled triangulation, reduced bias, and allowed for convergence and complementarity across data sources. This strengthened the credibility of the findings and offered more nuanced explanations of the phenomenon under investigation (16). By drawing on both qualitative insights (from firsthand accounts) and quantitative data (from historical EMT records), the study produced a rich, holistic, and comprehensive narrative of Ethiopia's emergency preparedness and response evolution.

To gather data, we first retrieved retrospective information on the activities and operations of the disaster management team prior to EMT, along with historical insights into national emergency response efforts (17). This involved reviewing available reports, surveys, periodicals, presentations, and bulletins accessed through the World Health Organization (WHO) EMT network and Ethiopian government websites. For information not publicly available, such as confidential reports, government presentations, and manuals, we contacted key informants involved in the disaster management team since the early phases of the emergency response. Additionally, we requested the National DMAT staff at the MoH to provide relevant local strategies, policies, and work plans.

Secondly, we conducted KIIs to gather additional qualitative insights. Participants were purposively selected based on their relevant expertise and direct involvement in the formulation, establishment, and implementation of Ethiopia's EMT. Priority was given to individuals who played key roles in emergency response planning and possessed firsthand knowledge of the EMT initiative, ensuring credible insights while helping to minimize potential bias. These key informants, referred to as “insiders”, included staff leading EMT components, focal persons from the MoH, National DMAT members, and other stakeholders engaged in EMT planning and response. In addition to their professional roles, these informants had access to crucial documents, including government reports, confidential presentations, manuals, and unpublished materials, offering essential insights into EMT development and operational challenges.

To collect qualitative data, 12 KIIs were conducted between December 2023 and April 2024. The semi-structured interviews facilitated in-depth discussions on the evolution of EMTs, policy frameworks, operational strategies, and encountered challenges. The interview guides focused on the EMT development process, the frameworks supporting their implementation, and the lessons learned (17). These interviews also served to verify findings and corroborate evidence from document reviews, ensuring a comprehensive and well-rounded analysis of Ethiopia's EMT initiative.

**Figure 2:**
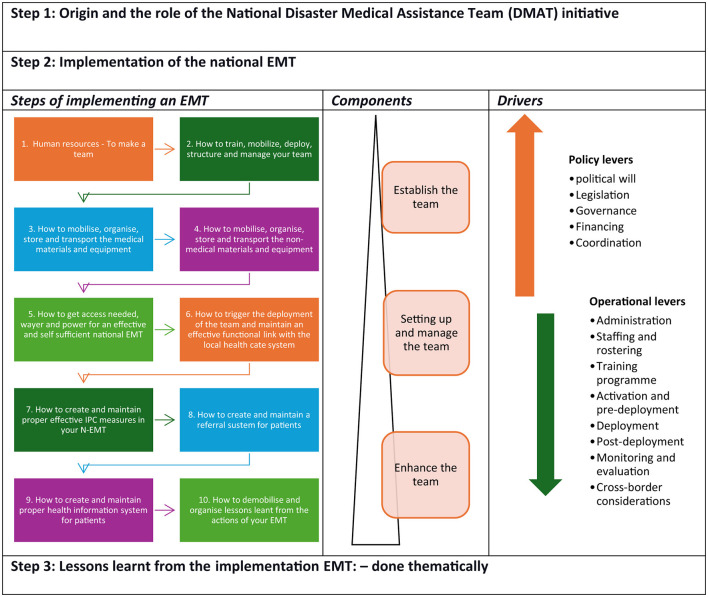
Framework of analysis [source: authors based on literature reviews (18)].

One researcher (BO) conducted interviews with participants during the study using a semi-structured interview guide. In this study, we systematically document and describe the formulation and implementation (development and progress) of EMTs in the Ethiopia in three sections. (a) tracing the origin of the emergency response work; (b) moving N-EMT based on the framework for implementing N-EMTs, targeting policy and operational levers (Figure 2) (18); and (c) describing the lessons learnt thematically (see Figure 2). The framework for implementing N-EMTs consists of 10 steps, each with a specific number of activities (Step 1: 6 activities, Step 2: 7 activities, Step 3: 5 activities, Step 4: 4 activities, Step 5: 5 activities, Step 6: 7 activities, Step 7: 3 activities, Step 8: 3 activities, Step 9: 2 activities, and Step 10: 6 activities) (18). In total, there are 48 activities for implementation. We used a scoring system to evaluate the achievement of each activity: 2 marks for total/full achievement, 1 mark for partial achievement (ongoing), and 0 marks for activities not yet commenced. The total cumulative score would be 96 if everything is fully achieved, indicating a fully implemented N-EMT.

All participants who agreed to undertake the interviews were informed of the study's purpose and gave verbal informed consent. The interviews were conducted in English and audio-recorded, lasting 30–45 min each. One researcher (BO) transcribed all the interviews verbatim and compared them to their audio files. All validated transcripts were extracted into MS Excel for ease of management and transparency of the analysis process. Data from document reviews and KIIs were synthesized using framework analysis, guided by the analysis framework (Figure 2). The lessons learned were analyzed thematically, following Braun and Clarke's framework (19).

## 3 Results

The results are presented in three sections. Section one presents the origin and the role of the National DMAT initiative and setting the movement to the EMTs. Section two presents the development of the roadmap for N-EMT in alignment with global EMT standards and the progress of the achievement/implementation of the N-EMT based on the roadmap while section three presents the lessons learnt from the implementation EMT.

### 3.1 Origin and the role of the national DMAT initiative

**Figure 3:**
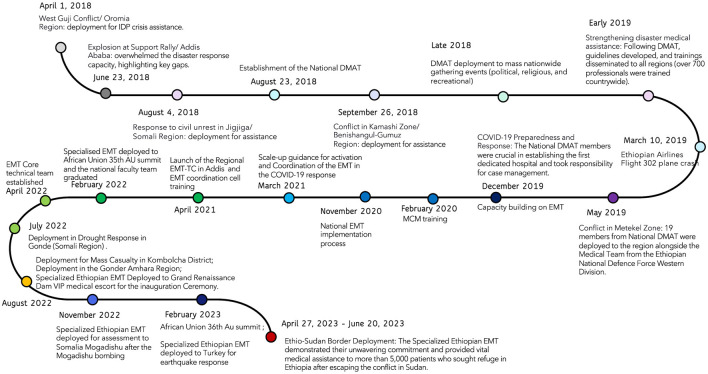
Historical landmark of Ethiopian Health Disaster Response.

The National DMAT initiative, established in August 2018, was created to provide structured medical support during natural and man-made disasters in Ethiopia, especially in response to increased conflicts. Prior to DMAT, public health emergency responses were unorganized and lacked coordination. The Emergency and Critical Care Directorate (ECCD) within the MoH developed DMAT by training health officers, nurses, emergency specialists, surgeons, anaesthesiologists, and general practitioners through a four-day intensive disaster management course. These trained professionals then trained regional counterparts, enabling DMAT teams to effectively respond to various emergencies, including public mass gatherings, conflict-related incidents, and the Ethiopian Airlines crash in 2019 (see Figure 3, Table 1).

**Table 1:** Summary of Ethiopian EMT deployment activities and events supported.

**Date/duration**	**Activity/event undertaken**	**Description of the activity/event**	**Number of patients seen**
Sunday, April 01, 2018	West Guji Conflict/Oromia Region	Different medical professionals from the Federal and Addis Ababa hospitals were deployed to support the crisis surrounding the creation of IDP from the event.	More than 1,000 patients
Saturday, June 23, 2018	Explosion at Support Rally/Addis Ababa	Medical professionals from Addis Ababa were deployed to support the treatment of hundreds of victims brought to hospitals in Addis Ababa following the explosion at a support rally.	40 patients
Wednesday, September 26, 2018	Conflict in Kamashi Zone/Benishangul-Gumuz Region	A team of medical professionals from federal hospitals was deployed to assist during the incident.	Hundreds of patients
Throughout 2018	Mass Gathering Events	Medical teams were deployed to various political, religious, and recreational mass gathering events nationwide to provide care in anticipation of potential mass casualties.	thousands of patients seen
Sunday, March 10, 2019	Ethiopian Airlines Flight 302 plane crash	The National DMAT played a crucial role as first responders (in comprehensive crisis response such as management of deceased individuals and the identification of victims) after the Ethiopian Airlines Flight 302 plane crash.	157 people
May, 2019	Conflict in Metekel Zone	A team of 19 members from the National DMAT was deployed to the region alongside the Medical Team from the Ethiopian National Defense Force Western Division.	300 patients
December, 2019	Onset of the COVID-19 Response	The National DMAT members were at the forefront during the beginning of the COVID-19 pandemic, played a crucial role in establishing the first dedicated hospital, and took responsibility for case management.	More than 200 patients
July, 2022	Drought response in Gode (Somali Region)	The Specialized Ethiopian EMT was deployed to the Somali Region to respond to the drought crisis in Gode with the primary objective of bolstering the surge capacity of the Gode pediatric ward, providing urgent medical care to those affected by the drought, community screenings for malnutrition and provided essential education to the local community. The team conducted specialized Clinical Management of Malnutrition training for those under 5 years old, further enhancing the region's response capabilities.	Clinical care to 99 SAM patients, 211 pediatric patients screened for malnutrition
February, 2022	African Union 35th Africa Union (AU) summit	Specialized EMTs were deployed to the African Union compound to provide emergency medical care for AU delegates.	85 patients
September, 2022	Internet Governance Forum	Specialized EMTs were deployed to the African Union compound to provide emergency medical care for the Internet Governance Forum delegates.	55 Patients
August, 2022	Mass Casualty in Kombolcha District	Specialized Ethiopian EMT was deployed (in a request triggered by the government) to mitigate mass casualties because of the conflict in the north of the county.	3,000 patients
August, 2022	Conflict in Gode Amhara Region	Specialized Ethiopian EMT was deployed (in a request triggered by the government) to mitigate mass casualties because of the conflict in the region.	2,500 patients
August, 2022	Grand Renaissance Dam VIP medical escort for the inauguration Ceremony	Specialized Ethiopian EMT provided VIP medical escort services at the Grand Renaissance Dam Inauguration Ceremony.	No Major Incidence
November, 2022	Somalia Mogadishu after the Mogadishu bombing	Following the devastating bombing, a specialized EMT was deployed to Mogadishu, Somalia. As part of a governmental mission, one member of the EMT participated in an official capacity to explore possibilities of cooperation in various emergency programs. After the tragic event, the team conducted a rapid pre-assessment and provided capacity-building support to enhance the local medical response capabilities.	Technical Support
February, 2023	African Union 36th AU summit	During the 36th AU summit, the specialized EMT set up a fixed and mobile Type 1 EMT station with two advanced ambulances and two basic ambulances at the African Union compound. 24 highly skilled EMT members were deployed for 1 week, ensuring prompt and expert medical support throughout the event.	More than 100 patients
February, 2023	Turkish earthquake event	A specialized EMT was deployed to Turkey for earthquake response. Collaborating with the National Search and Rescue team and other international EMTs (I-EMTs), they gained valuable experience and knowledge exchange, enhancing their disaster response capabilities.	No clinical service
April 27, 2023–June 20, 2023	Ethio-Sudan Border Deployment	The Specialized Ethiopian EMTs demonstrated their unwavering commitment and expertise during their deployment to the Ethio-Sudan Border following the Crisis in Sudan. Over this period, they provided vital medical assistance to patients who sought refuge in Ethiopia after escaping the conflict, delivered comprehensive emergency medical care, conducted rapid assessments, offered treatment for injuries and illnesses, performed surgeries, and provided essential psychosocial support.	Over 5,000 patients

The DMAT team made significant progress but identified several areas for improvement. Some of the challenges they faced included the lack of or insufficient development of standard operating procedures (SOPs), coordinating logistics and supply chains, and managing emergency finances. Stakeholder engagement was weak, with poor integration of clinical and public health responses and a lack of clear activation and incident command structures. Frequent deployments also strained human resource sustainability. To address these issues, DMAT prepared guiding documents, engaged in strategic planning, mobilized resources, and recruited experienced professionals. They standardized responses, conducted over 30 missions nationwide, and built capacity for 679 medical professionals and 80 health facilities across 12 regions. The Hub Hospital was designated for national coordination.

### 3.2 Roadmap development and implementation of N-EMT aligned with global EMT standards

In November 2019, Ethiopia conducted its first EMT workshop. Shortly after, the COVID-19 outbreak emerged, prompting the team to support the national response and case management within the Emergency Operations Center (EOC). Despite the challenges posed by the pandemic, induction training continued, and team members pursued specialized training abroad. To strengthen coordination, the MoH Emergency and Critical Care Directorate established a dedicated team responsible for overseeing responses, implementing EMT strategies, and developing a roadmap. In 2021, the team further advanced its capabilities by completing EMT Coordination Cell (EMT CC) training.

The progress and achievements of the roadmap are summarized in Table 2. As of the writing of this paper in June 2024, Ethiopia's N-EMT implementation score stood at 69 out of 96, reflecting its advancement toward full implementation. The breakdown by steps is as follows: Step 1–12; Step 2–11; Step 3–7; Step 4–7; Step 5–5; Step 6–11; Step 7–3; Step 8–6; Step 9–2; and Step 10–5. Overall, 27 activities had been fully implemented, 15 were partially achieved or ongoing, and 6 activities had yet to commence.

**Table 2:** Scope of the EMT.

**Task**	**Goal**	**Group of activities**	**Totally achieved (2)**	**Partially achieved (ongoing) (1)**	**Not achieved (0)**
1. Human resources—To make a team	**Detail**	**Activities**	**12**	**0**	**0**
	Identification of key members of the EMT, Multidisciplinary team (team lead, clinical doctors, nurses, Admin, Logistician, support staff); Depend on the focus of the EMTs	Identification and recruitment of the members of the EMT core management team (3 MoH officials- TL; Admin/HR; Technical person) and the 2 WHO officials (Liaison officer and OSL)			
		Identifying/clarifying the structure of the EMT core management team			
		Drafting the TOR for the functioning of the EMT core management team			
		Development of the roster of the teams (construction of the database composed of the *multidisciplinary team) (Team lead, clinical doctors, nurses, Admin, Logistician, support staff)*; Management of the tasks based on the key components around the emergency			
		Develop a joint work plan in accordance with the TORs, considering the three types of emergencies *(outbreaks, humanitarian crises, and natural disasters/mass casualties)*.			
		Work on the team's adaptability to each emergency *(outbreaks, humanitarian crises, natural disasters/mass casualties)*.			
2. How to train, mobilize, deploy, structure and manage your team	**Detail**	**Activities**	**8**	**3**	**0**
	Trainings for the EMTs, team management during “peace time”, roster, and team organization once deployed (architecture and log chart.)	Defining the types of training required based on the need (countries emergency context) (Coordination and management)			
		Establish the Organogram for the N-EMT members.			
		Working on the partnership with the international EMTs (I-EMT) (training, capacity building, procurement/donations of materials and equipment)			
		Management of the team: Draft Operational agreement for the functioning of the N-EMTs			
		Work on the modalities of deployment of the EMT team.			
		Identification of training items for standardization and assessment. Conduct training for the EMT team (in-country or regional)—*such as EMT induction training, Setting up training, and EMT field deployment training*.			
		Conducting the simulations/field deployment of the team for certification			
3. How to mobilize, organize, store and transport the medical materials and equipment	**Detail**	**Activities**	**4**	**3**	**0**
	Procurement, storage of drugs, tents, kits, PPEs, Medical materials….	Defining and procuring the package of medical materials and equipment needed			
		Defining and implementing the SOPs for the storage and management of the materials and equipment			
		Based on the context and opportunities, engaging partners for procurement and donation of materials.			
		Defining and implementing the SOPs for the transportation of the materials of the EMT			
		Defining the mechanism for ensuring the replenishment of the stockpiles [MoH & WHO Country Office (WCO)]			
4. How to mobilize, organize, store and transport the non-medical materials and equipment	**Detail**	**Activities**	**6**	**1**	**0**
	Access to food for the team and patients, transportation means, lodging of the team	Addressing how to meet the operational cost for the team (food and lodging) from WHO or MoH and Partners			
		Addressing the operational cost for the patients (Stabilization and management for 1 day; the target is referral ASAP)			
		Consider providing alternative lodging for the EMT members within the EMT facility (tents, beds) based on the country's context and the emergency.			
		Defining the transportation means of the EMT members during the mobilization, deployment and operating			
5. How to get access to needed water and power for an effective and self-sufficient N-EMT	**Detail**	**Activities**	**2**	**3**	**0**
	Generator, transportation or access to clean water (both for patients and team members)	Leveraging on existing sources—the system can be a surge in a hospital based on the available facilities in the area of the emergency. It could include a biomedical engineer (OSL member) on the surge team.			
		Identifying the power source (Generator, solar panel, or using existing local power system)			
		Identifying the possible water sources (Borehole/water truck); and ensuring the cleanliness and safety of the water.			
		Identifying the security mechanism of the team. The government to make arrangements for the security of the team.			
		Defining the mechanisms for the preparation and establishment of the EMT on the site (light onsite construction work; building of toilets; shower both for the patients and EMT members)			
6. How to trigger the deployment of the team and maintain an effective functional link with the local health care system	**Detail**	**Activities**	**8**	**3**	**0**
	Triggers for the deployment of the teams, who will take the decision, development of SOPs for the linkage with the local health system including with public health response systems	Defining the SOPs for the mobilization and to trigger the deployment of the team			
		Define the functional link between the EMT, local health facilities, and health systems.			
		Define the transportation means—Agreement with the regional county/district.			
		Plan for procurement and provision of internet and phones for communication (includes walkie-talkies)			
		Defining the mechanism for integrating the local health professionals/structures for the functioning of the EMT			
		Defining the linkage with the community—dedicating one member to link the team with the community. Using available systems and linkage with the surveillance/data manager for the facility/linking with the RCCE community (engagement with the community). Leveraging on the available resources			
		Engaging partners in supporting the deployment of the EMTs			
7. How to create and maintain proper effective Infection Prevention and Control (IPC) measures in your N-EMT	**Detail**	**Activities**	**2**	**1**	**0**
	Creation and maintenance of IPC measures, triage, and isolation rooms….	Define the PPEs to be used by the EMT team based on the type of emergency.			
		Defining other IPC measures to be implemented based on the type of emergency			
		Establishing the stabilization and isolation room (A tent to stabilize patient or a ward, crash carton) with all necessary IPC measures			
8. How to create and maintain a referral system for patients	**Detail**	**Activities**	**6**	**0**	**0**
	Identification of the referral pathways from the EMTs to other health care services	Defining the referral pathway, considering the nearby health facilities and the country context.			
		Defining the agreement mechanism for using the locally available transport means for patients to the closest hospital.			
		Defining the mechanism for leveraging the transportation of the teams under the surge project/including the transportation of the materials			
9. How to create and maintain proper health information system for patients	**Detail**	**Activities**	**2**	**0**	**0**
	Establishment/replication/extension of the health information management system of the local health system with the appointment of a dedicated data manager with the needed materials and resources	To avail the health information and patient reporting tools with the functional linkage with the local health system			
		Plan to have a dedicated team member in charge of the management and implementation of the health information system for the EMT.			
10. How to demobilize and organize lessons learned from the actions of your EMT	**Detail**	**Activities**	**4**	**1**	**0**
	Triggers for the demobilization of the team, who will decide to demobilize the team, and organization of the lessons learnt	Defining the SOPs for demobilizing the team (all in one document) based on the team members' maximum period and/or turnover.			
		Defining the specific steps and set of actions for clearing, cleaning and storage of the tents and equipment			
		Define the mechanism for providing psychosocial support to the team members during and after the deployment.			
		Plan for the communication and interaction with the communities and the local health authorities after the deployment			
		Plan and conduct the lessons learnt and undertake the documentation of the mobilization and deployment of the team.			
		Publication of relevant experience			

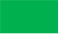
 = Totally Achieved; 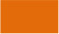 = Partially Achieved; 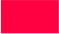 = Not Achieved.

#### 3.2.1 Step 1 and 2: human resource—to make a team, training, mobilizing, deploying, structuring and managing the team

##### 3.2.1.1 Initial planning and the composition of the core team

**Figure 4:**
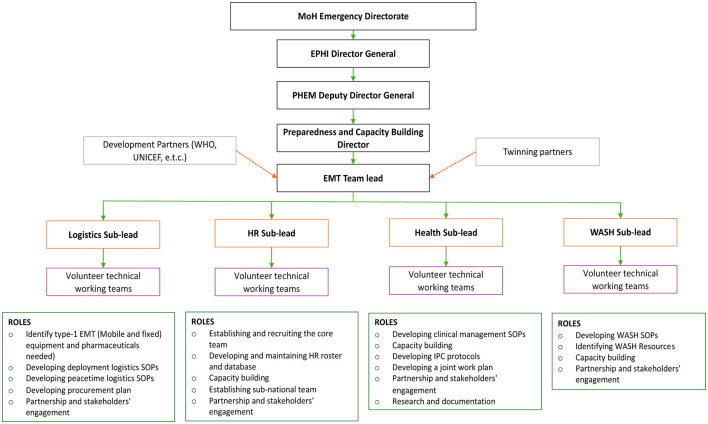
Organogram of the Ethiopian EMT.

At the beginning of the concept's introduction in the country, the team, with assistance from WHO Regional Office for Africa (AFRO) and WHO Country Office (WCO), planned a mapping exercise for strategy development. As part of this process, they identified the need for an EMT core technical team. Initially, this team was envisioned to include members from the MoH in roles such as administration/HR, technical support, coordination, liaison/information management, logistics, and 2 WHO support (comprising clinical and public health advisors, a logistics advisor, and safety and security and IPC advisors). As the implementation progressed through various deployments and government structuring, the structure of the EMT core technical team was confirmed. The core team consisted of a team lead and four sub-leads (in logistics, WASH, HR, and health) from the MoH, as well as 2 WHO support staff (a logistics advisor and a liaison officer) (see the organogram in Figure 4). Additionally, the team established formal links between the core technical team and the MoH governance system, ensuring coordination with the national incident management system (IMS).

##### 3.2.1.2 Team and the organizational structure

The EMT core technical team members' terms of reference (ToR) were established and aligned with the government's reporting arrangement. The team consists of a team lead and four other full-time employees, each responsible for a specific area. The team lead oversees overseeing the core team operations, establishing strategic links with the Ethiopian Public Health Institute (EPHI), and mobilizing resources. Each sub-lead manages a designated area, including the development of SOPs, work plans, and protocols, as well as capacity building, partnerships, and engagement efforts. Additionally, the health sub-lead has contributed to the formulation of research documentation elements, while the HR sub-lead has played a key role in developing and maintaining the team roster. To support these efforts, volunteer technical working teams assist each sub-lead, dedicating 2–3 h per week to their respective tasks. Figure 4 provides a detailed organogram outlining team roles and responsibilities.

**Figure 5:**
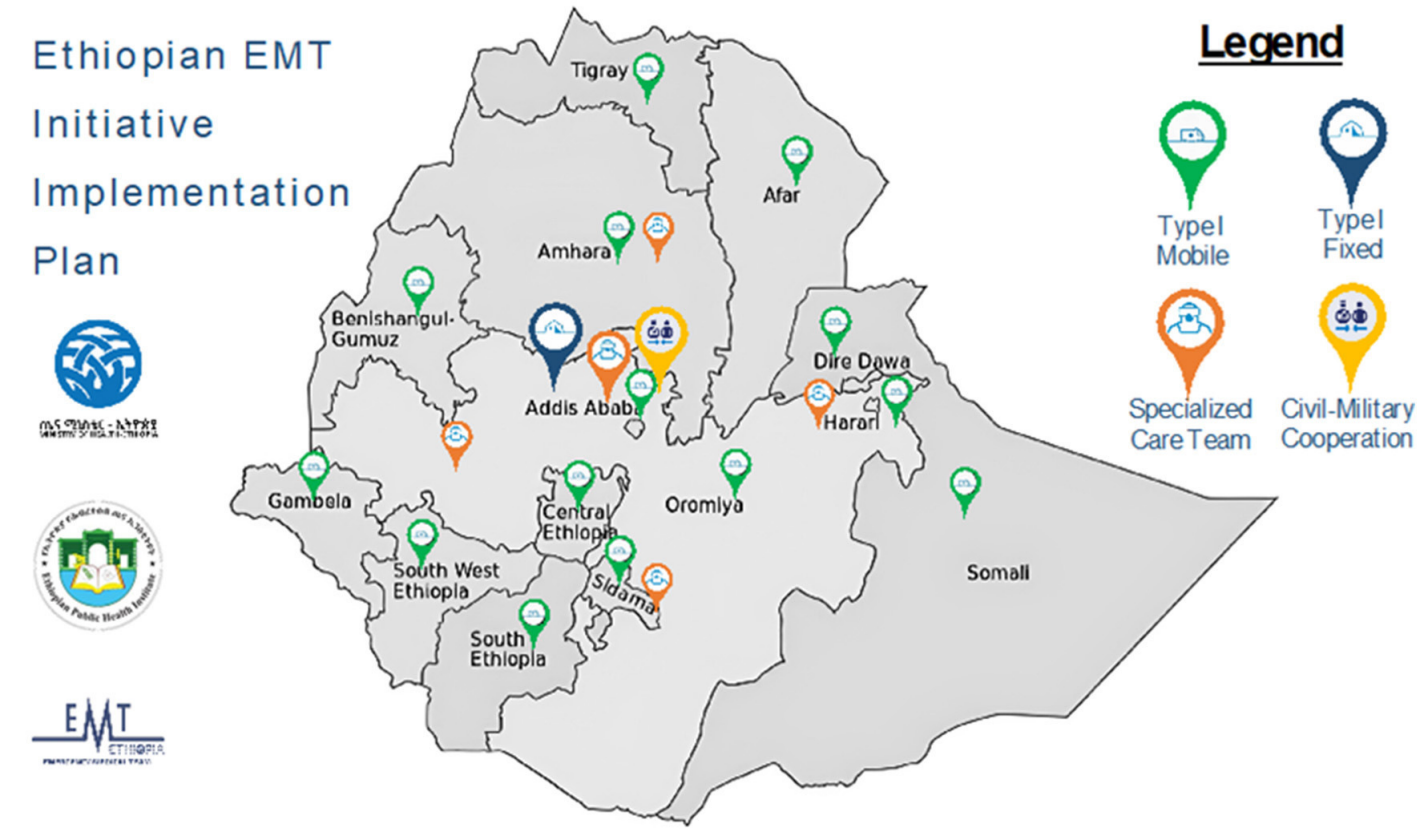
Envisioned national and subregional teams (source: the authors).

Looking ahead, the team has outlined a plan for developing national and subregional teams through the EMT initiative implementation plan. This plan envisions the establishment of 14 Type 1 mobile facilities, 1 Type 1 fixed facility, 5 specialized care teams, and 1 civil-military cooperation unit, as illustrated in Figure 5.

##### 3.2.1.3 Governance and coordination of the team

The governance and coordination of the EMT have evolved through multiple transition phases, allowing the team to assess different operational models and overcome challenges. Initially, the EMT functioned under the OCHA health cluster mechanism within the emergency department. Subsequently, coordination shifted to incident management systems and later integrated into the EOC structure, each phase being led by different directors. To establish a more stable and permanent framework, the EMT was ultimately positioned under the National Public Health Emergency Management (PHEM) system, which operates its own command center. This transition has strengthened emergency management capabilities under EPHI, ensuring a structured, sustainable working environment for the core technical team.

##### 3.2.1.4 Development of the team's roster

The administration and staffing of the teams are managed through various mechanisms. Under the leadership of the HR sub-lead, a database of multidisciplinary teams has been established, including clinicians, nurses, administrators, and logisticians. This effort has been ongoing alongside the preparation of SOPs and recruitment from different facilities. Deployments are made exclusively from this roster, which is currently maintained through an Excel-based system that remains inefficient. During the DMAT initiative, approximately 600 individuals received training, after which the EMT initiative took over roster management. To strengthen the team, additional personnel with a deeper understanding of EMT processes have been recruited from various facilities. Each deployment is structured based on emergency priorities and response needs, including outbreaks, humanitarian crises, natural disasters, and mass casualties. The roster includes multiple cadres of health workers, with increased emphasis on rehabilitation and psychosocial support to ensure balanced team distribution. Newly recruited staff must undergo induction training to familiarize themselves with EMT concepts before deployment. The HR roster is being developed in collaboration with partners, and the Ethiopian EMT recruitment, selection, and roster management SOP has now been finalized.

##### 3.2.1.5 Development of a joint work plan

To ensure effective response operations, a joint work plan was developed based on national response needs and Vulnerability Risk Assessment and Mapping (VRAM). This plan has guided the implementation of key components, ensuring alignment with priority areas.

##### 3.2.1.6 Administration and staffing

The team relies on existing government institutions and recruitment procedures, streamlining the process of designing a compensation mechanism. Government systems have facilitated background checks through pre-existing records and document/certificate reviews. Recruitment efforts have been conducted through campaigns, professional associations, advocacy meetings, and platform exhibitions. Candidates are required to submit an official CV and an expression of interest, followed by interviews and selection. The selection process includes document reviews and evaluation of performance during interviews. To enhance the selection process, the team is refining interview questions to incorporate psychosocial elements, testing candidates through scenario-based assessments to evaluate their ability to handle response challenges and stressful situations. Those selected are required to complete an induction course, with the goal of ensuring 100% training coverage for all team members before deployment.

##### 3.2.1.7 Training

The team has provided trainings, simulations, and field deployments tailored to new members' needs. A summary of the trainings is presented in Table 3. Regional training sessions have been conducted face-to-face, though this approach can be costly. These sessions have been made possible with support from donors, including WHO AFRO, WHO HQ, WHO Academy, UK-MED, and PCCM. Additionally, the Ethiopian EMT Training Package has now been finalized, ensuring standardized capacity-building for EMT personnel.

**Table 3:** A selection of the trainings conducted by the regional faculty.

**Training**	**Date**	**Number of Participants**	**Remarks**
Mass casualty management			Training conducted by the WHO Academy at the EMT Training Center (TC)
EMT TM induction	03/22, 06/22, 07/22	3	Training conducted at the EMT-TC
10-step implementation	03/22	1	
Case management mentorship	09/23	1	
Case management workshop	09/23	1	
Burn management specialized training	10/22	1	
EMT ToT	02/22	1	

Rehabilitation training was planned for October in Ethiopia but has not yet been completed. Surgery in a humanitarian environment was also planned but has not yet happened.

##### 3.2.1.8 Deployment

The deployment process begins with activation. Given Ethiopia's decentralized political system, where each region has autonomy, emergency response efforts start with communicating the situation to the relevant regional authorities. Once an emergency is detected, the team informs the concerned regions, which then submit a formal request for assistance. This request is forwarded to higher officials, who prepare a proposal or concept note outlining OSL requirements, human resources (HR) needs, deployment duration, composition, and budget. Once approval is granted, the team immediately contacts members, organizes pre-deployment orientations, conducts team-building activities, and facilitates physical introductions.

Next, the logistics phase is initiated, during which medical and non-medical supplies are mobilized from the team's warehouse. When a deployment request is received, the team first identifies available staff from the roster and confirms their availability. Once an individual accepts, their respective health facility (typically a government department) is notified via an official request letter to temporarily release them for deployment. These facilities continue paying the staff's salary during deployment. In response, the health facility provides either a verbal confirmation or an official letter, which is then shared with the deployed team.

The compensation structure for deployments is defined by the government but is often considered inadequate. Each deployed individual receives a Daily Subsistence Allowance (DSA) of USD 10 per day, with a typical deployment lasting 30 days. This period includes 3 weeks of active work, 3–6 days of travel (depending on the deployment location), and 2–3 days post-deployment for recovery. In some cases, partners such as WHO provide additional support, covering accommodation and extra costs beyond the 30-day period.

A well-defined team structure is established before deployment, assigning specific roles such as team lead, deputy team lead, data lead, clinical operations, clinical leads, documentation lead, pharmaceutical, logistics, Infection Prevention and Control (IPC), and Water, Sanitation, and Hygiene (WASH). Once deployed, teams engage in daily debriefings to reflect on lessons learned.

##### 3.2.1.9 Coordinating multiple teams

Deployments often involve multiple teams working in succession. While one team is in the field, preparations for a second team are already underway. The second team is carefully selected and trained to ensure a seamless transition, with one or two individuals from the first team remaining to oversee ongoing operations. Given the complexity of coordinating multiple teams, clear communication and coordination are essential to ensure success.

#### 3.2.2 Steps 3 and 4: mobilizing, organizing, storing and transporting the medical and non-medical material and equipment

##### 3.2.2.1 Pharmaceuticals

The EMT collaborates with the Ethiopian Pharmaceutical Supply Service (EPSS), a dedicated agency responsible for medical supply distribution. Each year, a renewable Memorandum of Understanding (MoU) is signed between the hosting institute (EPHI) and EPSS to formalize the partnership. EPHI operates approximately 20 warehouses nationwide, which support deployments by serving as supply hubs. Before each deployment, the team compiles a list of required supplies and equipment, submits it to EPSS along with payment details, and requests direct delivery to the deployment site. The nearest warehouse dispatches the supplies, reducing logistical burdens such as transportation, warehouse, and stock management.

This supply system has been tested in five deployments and has proven effective; however, efforts are ongoing to improve timeliness. When a written request is submitted, supplies are delivered without requiring advance payment. Once the team arrives at the deployment site and prepares the operational area, the requested medications typically arrive within 2 to 48 h. However, the team acknowledges that full fulfillment of orders is not always guaranteed. On average, 70% to 80% of requested supplies are provided, requiring contingency planning to address shortages. Partnerships with agencies like WHO and UNICEF help fill these gaps by providing kits, supplements, and medications. The team shares a medical request form with these organizations, and upon confirmation, the requested supplies are delivered directly to the deployment site. The goal is to establish a standardized warehouse within the field hospital to store essential medical equipment and ensure readiness for future deployments.

##### 3.2.2.2 Equipment

The EMT Training Center (TC) operates two warehouses, one of which is owned by WHO. These warehouses store tents, chairs, wheelchairs, stretchers, and other essential equipment. During deployments, the team submits a supply request to WHO via email, prompting the preparation and transport of the required equipment to the designated location. Additionally, the EMT TC manages a separate warehouse adjacent to WHO's, which contains tents, beds, trauma kits, lights, and other supplies. Both warehouses play a critical role in ensuring rapid deployment and efficient emergency response.

To facilitate transportation, the required equipment is identified and moved closer to the airport for air transport. The EMT TC's strategic location near the airport allows for quick mobilization of supplies and personnel via flights. To enhance storage capacity and preparedness, the team recommends allocating government or partner budgets for constructing permanent standardized warehouses within the compound. Currently, standardized tents (larger MSU tents) are used as makeshift warehouses, but improvements are needed to enhance storage efficiency.

To streamline operations, the team is developing SOPs to ensure all essential supplies are consistently available during deployments. However, shortages of critical items like gloves and masks occasionally occur. As a preventive measure, the team prioritizes thorough pre-deployment inventory planning to mitigate supply gaps and maintain operational readiness.

#### 3.2.3 Step 5: getting access needed and access to water and power for an effective and self-sufficient N-EMT

*The team has relied on existing systems since its inception*. During deployments, they borrow generators and water locally, making daily payments as needed. Drinking water has been acquired through various methods, including bringing their own supply or chlorinating water at the deployment center. Recently, the team obtained a mobile clinic equipped with its own generator, which served as a power source during the latest deployment. This clinic includes pharmacy and laboratory sections for basic bedside testing, such as random blood sugar tests and non-infectious sample management.

Additionally, the team has been provided with two advanced ambulances. Before deployment, significant time is spent defining mechanisms for setting up the EMT site, including on-site lighting, construction work, and the installation of toilets and showers for both patients and EMT members. However, since team members were not required to stay overnight in their camp during deployment, they were accommodated in hotels, reducing the need for overnight lighting.

##### 3.2.3.1 Security

For security, the team ensures proper clearance before each deployment. The team lead coordinates with ground teams in advance to confirm security readiness. For instance, during the Metema deployment, rumors of security threats along the main road prompted the team to arrange a military escort. Upon arrival at the airport, the military patrol met the team and escorted them to the operational site. Furthermore, UN-BSAFE training has now become a mandatory requirement for all Ethiopian EMT team members before deployment, ensuring preparedness for security challenges.

#### 3.2.4 Step 6: trigger the team's deployment and maintain an effective functional link with the local healthcare system

*The team has been utilizing an existing system while also introducing a new working approach*. Through their experience in handling complex emergencies, they have learned to integrate their operations with the country's existing health and coordination systems. During deployments, they identify a backup hospital for patient referrals, sometimes located up to 50 km away. This referral system improves patient flow, reduces facility overload, and ensures continuity of care. As a result, it has been incorporated into the referral SOP, with all team leads responsible for designing and initiating the referral linkage.

*To strengthen coordination with local communities, the team established structured mechanisms that enable dedicated members to collaborate with both community representatives and central management*. Coordination during deployments has been managed at two levels: between senior officials at the MoH and regional or local community leaders, and within the team itself to ensure smooth execution of activities and achievement of deliverables. A strong example of effective coordination was observed during the malnutrition management deployment in the Somali Region, where all team members were well-aligned, informed about their tasks, and highly motivated. However, challenges were encountered during deployment in Kombolcha, Amhara Region, where coordination between the local and deployed teams was insufficient. The regional teams were not properly received, guided, or integrated into the operations, leading to communication gaps between the MoH, hospitals, and regional authorities. Issues such as unclear movement protocols, team living arrangements, and facility setup further hindered efficiency.

*Additionally, the team has been leveraging available systems and establishing links with facility surveillance/data managers by assigning a dedicated member to compile and manage daily deployment data*. However, data collection remains paper-based rather than software-driven. The team also engages with partners to support EMT deployments and typically establishes community engagement linkages prior to deployment, but this approach has not always been effective. To ensure consistency, formalization of this process through an SOP is needed.

#### 3.2.5 Step 7: creating and maintaining proper effective IPC measures in the N-EMT

The team has a technical working group responsible for developing WASH and IPC standards. They are actively working to implement these standards. Additionally, they are focusing on safeguarding, which has been included in the country's training curriculum. Efforts are underway to ensure that all personnel understand and adopt IPC protocols, maintaining the quality of care required.

#### 3.2.6 Step 8: creating and maintaining referral systems for patients

The team's referral pathway is not flawless, but they work closely with local regions during deployments to identify nearby health facilities capable of managing patients beyond their capacity. This requires an understanding of the locations of available referral facilities. Additionally, they have an agreement with the hospital transport system to ensure patients are transported to the nearest suitable hospital using locally available transportation. For example, in one instance, patients traveled 100 km to a facility despite a suitable one being just 50 km away. Meanwhile, the transportation of materials and personnel for the surge project remains ongoing.

#### 3.2.7 Step 9: creating and managing proper health information for patients

*The team is actively digitizing its operations, focusing on four key areas: roster management, capacity building, information management, and logistics supply*. Among these, roster management and capacity building are the top priorities. To replace printed copies previously distributed to each team member, the team has introduced the Minimum Data Set (MDS) tool, which enables the recording of patient information in Excel every night. This recorded data is then shared with existing health facilities upon the team's departure. Rather than relying on hardcopies, efforts are underway to fully digitize data capture and develop dashboards for improved accessibility and efficiency.

During deployments, one team member is assigned to collect data on referrals, prescriptions, and consumables. Currently, the MDS tool is used manually, requiring printed copies for each patient, leading to an accumulation of up to 6,000 paper records. To address this, the team aims to develop digital tools that will simplify and streamline data collection and management, though internet connectivity challenges sometimes hinder progress.

In addition to digitization efforts, the team is also working on producing a magazine and report documenting recent deployments, challenges, and lessons learned. To recognize team members, a brochure featuring their portraits has been created as both a token of appreciation and a keepsake. The MoH provides technical support, while the team collaborates with the African Union and the Ministry of Foreign Affairs to further enhance digital information management.

#### 3.2.8 Step 10: demobilizing and organizing lessons learnt from the actions of the N-EMT

##### 3.2.8.1 Debrief and post-deployment

At the end of each day's deployment, the team holds a mandatory 30-min debriefing session at 4:45 PM. During this meeting, each member shares challenges encountered and discusses the data collected. These discussions can sometimes become emotional and may lead to prolonged debates as team members process their experiences.

Following the deployment, psychiatrists facilitate a group debriefing session, which includes counseling and a retreat to help the team process their experiences and manage stress and emotional impact. After these sessions, the team lead and the MoH organize a recognition ceremony, presenting certificates of appreciation to participants and partners in acknowledgment of their contributions to the response.

## 4 Lessons learnt from the implementation EMT

### 4.1 Political support and government commitment to the EMT concept

Ethiopia's EMT initiative has received significant political support from high-level government offices and international partners. The MoH officials were highly receptive to the concept, with leadership recognizing its necessity and making a concrete commitment to its implementation. The timing of the EMT's introduction was seen as a strategic opportunity to strengthen the country's emergency response capacity, given the multiple crises it was facing.

A major milestone in this process was the launch of the EMT TC on April 14, 2021, jointly established by the WHO and the Ethiopian MoH. The launch coincided with the introduction of the EMT concept, and the presence of top government officials at the event generated significant political interest and media attention, further solidifying national acceptance of the initiative.

The Ethiopian government's high-level political and financial support, including direct involvement from the Prime Minister's office, played a crucial role in enhancing the N-EMT deployment to Turkey, fostering national solidarity, and reinforcing the EMT's credibility through international collaborations and emergency response efforts:

“*…. For example, during the N-EMT deployment in Turkey, the top leadership provided the needed support (financial and political) to enhance the deployment. The concept note drafted by the team was approved promptly because it provided an opportunity to learn from the other 550 teams deployed and the historical diplomatic relations between the government and Turkey. Even the Prime Minister's office was involved during the deployment, tweeting the progress of the N-EMT's deployment to Turkey almost daily. This showed good solidarity and overwhelmingly made the population want to join the EMT response team. The team has joined a team organized by the Prime Minister's in visiting the Mogadishu blast areas following the blast because of their expertise. They even participated in the distribution of food and assessments on the ground.”—**Respondent 01***

Beyond individual leadership support, government commitment to emergency preparedness and response has been institutionalized. The EMT framework is now embedded within the government's system and structure, ensuring that its progress continues regardless of changes in leadership. This institutionalized approach reflects a long-term commitment to strengthening national emergency response capabilities, reinforcing the EMT's sustainability within Ethiopia's broader health and disaster response framework.

### 4.2 The EMT has expanded its focus areas beyond traditional response work and has improved its capabilities through multiple deployments

The EMT has expanded its focus beyond traditional response efforts, enhancing its capabilities through multiple deployments in mass gatherings, conflict zones, malnutrition response, and high-level meetings such as African Union summits, leveraging Ethiopia's strategic location (see Table 1). These deployments have not only increased the team's visibility but also played a key role in redefining and shaping their roles and activities. Each mission has demanded diverse technical expertise, contributing to the team's growing technical proficiency and interpersonal experience.

### 4.3 The alignment and focus of the coordination mechanism under the EPHI have improved operations and collaborations

The alignment and strengthened focus of the coordination mechanism under the EPHI have significantly enhanced operations and collaboration. This has been operationalized through the formal inclusion of the Ethiopian EMT's role in EPHI's powers and duties, as outlined in the Regulation presented to the Ministerial Council in February 2023.

One key improvement has been the leveraging of existing agreements and collaborations to facilitate access to essential resources, such as medications and medical professionals, during deployments. This approach has streamlined bureaucratic processes, enabling faster decision-making while also increasing stakeholder involvement and commitment to the initiative. Strengthened EPHI partnerships have allowed RRTs to assist EMTs by performing pre-deployment evaluations, enhancing readiness, coordination, and integration with the health system:

“*…. Collaborations have also enabled teams under the EPHI, such as the RRTs….focused on surveillance, to work as an advanced team for the EMTs before deployments to assess the situation and conditions on the ground. This is in line with the context that the EMTs support the existing health system in providing emergency services.”—**Respondent 03***

### 4.4 There has been strengthening verification, HR, and logistics through twinning partnerships

Through twinning partnerships, the team has collaborated with UK-MED and PCPM to enhance the verification process, as well as HR and logistics arrangements. Both partners have already passed the verification phase and have shared their experiences and best practices with the team.

#### 4.4.1 PCPM's contribution: logistics and infrastructure

PCPM has played a key role in strengthening logistics and operational planning. Their support included purchasing five 50 m^2^ tents, response vehicles customized with team-specific content and branding, trauma kits, and stretchers. PCPM also contributed to the layout design and safety/security communication, developing two SOPs for layout configuration. One SOP provided detailed specifications for room layouts, including the command-and-control center, delivery rooms, waste management, water and electric distribution, emergency exits, fire emergency protocols, infectious and non-infectious areas, and mapping of clinical and non-clinical zones. Additionally, PCPM introduced two SOPs on internal communication, having previously procured 12 communication radios to enhance the team's operational coordination.

#### 4.4.2 UK-MED's contribution: HR recruitment and training

UK-MED's collaboration focused on HR recruitment and selection processes. Since the team initially relied on paper and Excel-based systems, UK-MED introduced them to web-based HR and training platforms, allowing for cost-effective, accessible, and manageable learning solutions. This exposure helped the team redesign their learning platform, ensuring greater efficiency. UK-MED also provided training in resource mobilization and invited team members to participate in their reclassification simulation exercise in Manchester. Two team members traveled to Manchester, where they gained firsthand experience in warehouse management, demobilization, donation handling, and professional conduct within emergency response operations.

“*…. Since the team's work was initially paper and Excel-based, they learned about UK-MED's web-based platforms for HR and training, which were conducted through web-based training. This helped the team to clearly understand how to design their learning platform so it's cost-effective, accessible, and manageable.” –* ***Respondent 01***

These collaborations have significantly enhanced the team's operational capacity, ensuring improved verification, streamlined logistics, and more efficient HR processes tailored to Ethiopia's context.

### 4.5 Innovative funding strategies contribute to successfully implementing the EMT

The financing of the EMT has been integrated into emergency planning, grant writing, and national planning systems. According to Respondent 02, EMT funding has been incorporated into current EPI annual plans and programs as well as into collaborative initiatives with partners such as Africa Centers for Disease Control and Prevention (ACDC), United States Agency for International Development (USAID), the World Bank, the Global Fund, the Pandemic Fund, and African Volunteer Health Corps and the Strengthening and Utilizing Response Groups for Emergencies (AVOC-SURGE). In addition to the government's contribution, additional funding has been secured through these partnerships.

“*For example, it has been included in the current EPI annual plans and programs and in plans with partners such as ACDC, USAID, World Bank, Global Fund, Pandemic Fund, and AVOC-SURGE…… funds have been received in addition to the government's contribution.”—**Respondent 02***

With the available funding, the team is prioritizing regional team building over national team building. For subnational teams, the focus is on mobile teams, whereas at the national level, the emphasis is on Type 1 teams. The team anticipates securing a dedicated budget in the upcoming fiscal year. During the transition period, they relied on MoH and EPHI budgets to support EMT operations.

### 4.6 Emergency response is always prioritized over preparedness, which has been the case in Ethiopia

In Ethiopia, emergency response has consistently taken precedence over preparedness. However, past emergencies have demonstrated a pressing need for stronger preparedness initiatives. The country's recurring humanitarian crises have highlighted the urgent necessity of ensuring resources are readily available for effective emergency response.

For instance, during the internal war, the response team was understaffed and lacked the time required for adequate training and preparation. To compensate, virtual meetings were held, and individuals with specific communication skills were recruited to report to the central command. This experience underscored the need for continuous recruitment, as time constraints often prevent teams from receiving sufficient preparation before deployment:

“*For example, during the internal war, the team was understaffed and lacked time to train or prepare. Virtual meetings were conducted, and individuals with specific communication capabilities were recruited to report to the central commander.”—**Respondent 02***

The incident management structure for conflict response includes a talent search unit, which is responsible for deploying teams to different parts of the country as needed. Additionally, peripheral governments submit requests to the central government to ensure an effective and coordinated emergency response. Strengthening preparedness efforts alongside emergency response remains critical for improving Ethiopia's overall disaster management capacity.

### 4.7 There is over-reliance on the EMT team and expectations for equipment retention during deployments

During deployments, the EMT team often faced over-reliance from local health systems, with some regions depending heavily on their support instead of utilizing existing local capacity. This dependence extended beyond their designated tasks, as they were frequently expected to provide additional services, such as training personnel, repairing equipment, and reorganizing facilities. A clear example occurred during a mission in the Somali region, where the EMT team was expected to train ICU staff and repair medical tools that local personnel had been responsible for maintaining. In the pharmacy sector, although supplies were available, the lack of proper organization hindered efficiency, prompting the EMT team to step in and streamline inventory management:

“*During the mission in the Somali region, the system relied heavily on the team to work and carry out their tasks. An example of this is when the system depended on the team to provide training on ICU work and to fix tools that they had previously been responsible for. In the pharmacy area, the team helped organize the pharmacy. They found that the pharmacy had plenty of items but was not organized. As a result, the team had to step in and organize the items in a more streamlined fashion.”*—***Respondent 03***

Additionally, the EMT team often encountered expectations to leave behind their equipment after completing their mission. This was fuelled by a perception that, given their visibility in media, association with the Prime Minister's office, and proximity to central resources, they had unlimited access to supplies. Many regional officials assumed they deserved equal material support and viewed the EMT team as a source of ongoing provisions.

A significant instance of this occurred during the Sudan crisis, where the team had to decide on the most strategic point of entry (PoE) for deployment. With multiple border crossings receiving an influx of people fleeing Sudan, they selected Metema, as it was the busiest and deemed the most critical location for intervention. However, the overwhelming number of displaced individuals created logistical challenges, and local health officials began questioning the deployment strategy and resource management. Due to insufficient resource advocacy, the EMT team was ultimately compelled to leave behind essential equipment, including refrigerators, wheelchairs, walkers, and examination beds. This decision led to tensions, with a neighboring region filing a formal complaint about the perceived inequitable distribution of aid.

### 4.8 Implementing advanced teams and enhancing pre-deployment preparation

The concept of advance teams was introduced in response to lessons learned and complaints from previous deployments, highlighting the importance of thorough preparation before deployment. Given that team members are often geographically dispersed, effective pre-deployment planning is essential for ensuring smooth adaptation to new environments and maintaining operational efficiency.

An advance team consists of experts deployed ahead of time to assess the situation and gather critical information about the area of response. This proactive approach has proven effective in resolving political crises and governance issues, ensuring that teams are well-prepared before full deployment. While emergency deployments require rapid response, adequate preparation time remains crucial for success. Each team member must adjust to the new environment and ensuring the presence of well-prepared personnel with the right attitude, soft skills, and a shared understanding is key to mission effectiveness.

However, communication challenges between teams, as well as individual attitudes and commitment levels, can create obstacles. If some team members are not fully aligned or less committed to delivering quality service, it can place a burden on others. Therefore, effective communication—both within the team and with healthcare workers and local communities—is vital for seamless collaboration.

Adapting to the deployment environment can also be difficult, especially for individuals unfamiliar with EMT operations, requiring flexibility and adaptability in diverse situations. Deploying female team members to remote areas presents additional challenges due to high demand, as they must often balance multiple personal and professional responsibilities while responding to urgent deployments.

### 4.9 Preparation before deployment is also critical, as each team member may not be close to one another and will need to adapt to the new environment.

While emergency deployments require a rapid response, adequate preparation time is essential for success. Having the right people with the appropriate attitude, soft skills, and a shared understanding is crucial during deployment. However, communication challenges and individual attitudes can create obstacles. When team members are not aligned or fully committed to delivering quality service, the burden falls on others, impacting overall performance. Effective communication among team members, healthcare workers, and communities is therefore critical. Additionally, adapting to new environments and deployment areas can be difficult, particularly for individuals unfamiliar with EMT operations who must remain flexible and adaptable. Deploying female personnel to remote areas presents additional challenges due to demand, requiring them to quickly and efficiently balance various personal and professional responsibilities to meet urgent deployment needs. As noted from the following example from the field:

“*…For example, there were only two females during our deployment in Matama. Because of the demand, having females deployed in remote areas of the country is very challenging. For example, one female employee was told about one of her deployments on Wednesday morning and was on the ground on Thursday. Because of the urgency of the deployment, healthcare workers need to manage different aspects of their life. To leave your facility that way, you're leaving a comfortable life and going to a remote area, maybe some places you're completely unfamiliar with, and you may encounter different cultural issues.”—**Respondent 05***

### 4.10 Strengthen coordination, communication and prioritization to improve EMT deployment

At the national level, coordination is generally effective, supported by comprehensive training and orientations before deployment. Since professionals come from diverse hospitals and organizations, each with its own culture and work habits, pre-deployment workshops help align strategies and ensure a cohesive approach. While coordination with health facilities is maintained, teams on the ground sometimes feel isolated, as national teams often operate independently. This disconnects partly stems from the perception that nationally deployed teams receive better financial packages, despite Being volunteers. To address this, discussions should be held to harmonize team orientations with facility operations, fostering better integration. Additionally, deployment and communication skills should be incorporated into training programs, as misunderstandings frequently arise due to communication gaps or inadequate communication skills. Emphasizing professional and clear communication from the initial capacity-building stage can help mitigate these challenges.

Furthermore, differences in situational understanding between the team in Addis Ababa and those on the ground can create obstacles. Establishing a clear communication channel is essential to ensure national teams accurately comprehend field conditions, visualize challenges, and provide appropriate support. Coordination gaps also emerge when dispatching additional personnel and resources to deployment sites. To prevent this, it is crucial to communicate ground realities effectively to all team members involved in decision-making and operations.

In some recent deployments, high demand for injection medications led to long queues and crowding at the deployment center. To manage this, the team collaborated with a nearby health facility to refer patients needing wound care and injections. Ensuring clear communication among healthcare workers, patients, and response teams is vital for smooth operations. At the end of each deployment, the team reviews priorities, revises medication lists, ensures a seamless transition, hands over data to nearby facilities, donates equipment, and provides additional capacity-building support. Since some deployments can last up to 9 months, it is important to prevent local systems from becoming overly reliant on EMT teams and deferring all responsibilities to them.

*Psychological support and debriefing sessions are always provided to teams following a response*. For MBT team members, this is facilitated by a psychiatrist, while in other deployments, post-debriefing sessions are conducted for immediate team members. Additionally, certification is provided as part of the post-deployment process.

## 5 Discussion

This paper describes the progress of Ethiopia's emergency response mechanisms and the development of its N-EMT. It highlights the lessons learned from implementing the N-EMT, which can be used by other teams implementing their N-EMTs.

Our findings indicate that the DMAT initiative, established in August 2018, aimed to enhance response to a rise in man-made emergencies, including conflicts, requiring deployments to peripheral regions. It introduced a structured and effective response framework with trained professionals for medical crises (20). The initiative was strategically restructured to address diverse response challenges, leveraging Ethiopia's unique challenges and historical emergency response developments, leading to the creation of EMTs. This development produced a roadmap for N-EMT in alignment with global EMT standards and addressed gaps highlighted by the COVID-19 outbreak, as also postulated by the other studies (21). This finding strengthens the other research finding that EMTs are crucial for clinical and public health emergency responses, as evidenced by investments in deployable health emergency capacity by other countries, demonstrating their ability to mobilize professional EMTs for timely, self-sufficient responses to diverse emergencies and reducing reliance on international aid (22).

Further, our finding shows that a core team was established to coordinate EMT response efforts, and it was modified as needed based on planning and response requirements. Having a team to set the agenda for EMTs is imperative, as previous research indicates that uncoordinated medical teams without a proper agenda can significantly disrupt national emergency plans, deplete resources, and result in unnecessary disabilities and deaths (23). Additionally, the MoH guided the initial planning and team composition, aligning roles and governance structures. This alignment showed that, government coordination and support are crucial to ensure EMTs have the necessary resources, including medical supplies, equipment, and personnel, to deliver optimal care in resource-limited environments. Researchers have also shown that governments play a crucial role in the development of EMTs (24). This reliance on the government has allowed the EMT to strategically adopt a collaborative approach with the government and even partners to manage, mobilize, organize, store, and transport medical and non-medical materials, ensuring adequate pharmaceuticals and equipment for various responses. They strategically relied on existing government systems, such as operational warehouses and medical supply chains, while securing access to essential resources like water and power for self-sufficiency. Besides, additional support is being provided through the development of a roster of response staff in view of the jointly developed work plan, which includes administration, staffing, training, and deployment procedures.

Further findings show that the team developed strategies for deployment and integration with the local healthcare system, and coordinating with the community through a mechanism that involved dedicated team members working with locals and central management. The team utilized existing systems and introduced new ones, ensuring daily data compilation by a designated member and maintaining effective IPC measures. They also created a referral system for patients and maintained proper health information. Post-deployment, they conducted debriefings and incorporated lessons learned. These findings align with other literature suggesting that effective EMT functioning relies on interactions among stakeholders, including national governments, international organizations, non-governmental organization (NGOs), local agencies, community stakeholders, and the private sector, which are crucial for enhancing EMT coordination and integration through systematic classification and registration, national stewardship, community engagement, and research and data collection (24).

Our findings have further shown that, the EMT in Ethiopia has received significant support from high-level government offices and partners, which has been crucial to its establishment and success. Officials from the MoH have demonstrated strong commitment to emergency preparedness and response, ensuring long-term sustainability and integration into the government system. This institutional support goes beyond individual leadership interests, with ongoing political backing facilitating multiple deployments and enhancing the EMT's effectiveness and resilience. This support highlights the importance of political will in advancing national health emergency frameworks and improving operations and collaborations through coordination mechanisms under the EPHI.

The N-EMT has broadened its operational scope beyond conventional response activities, aligning with global trends in disaster response diversification. By deploying teams for mass gatherings, conflicts, and malnutrition responses, the N-EMT has enhanced its operational capabilities. Participation in high-level meetings, including those of the African Union, underscores Ethiopia's strategic engagement in regional health diplomacy. Collaborative efforts with organizations such as UK-MED and PCPM have significantly refined the EMT's verification processes, human resource management, and logistics through twinning initiatives, consistent with practices observed in other I-EMT collaborations. Additionally, innovative funding strategies emerging from these partnerships have been crucial for the EMT's sustained and effective implementation, reflecting a broader trend in emergency medical funding strategies.

The deployment of Ethiopia's EMT has provided valuable lessons, refining their operations. Excessive regional reliance on the EMT, expecting them to leave equipment behind, underscored the need for clear boundaries and resource management. The use of advance teams, experts sent ahead to assess and gather information, has enhanced preparedness and situational awareness. Adequate preparation time, despite emergency urgency, is crucial for effective deployment, ensuring team adaptability. Effective coordination and unified communication are essential, though advocacy and communication about EMT roles need improvement. Prioritizing immediate care areas and providing psychological support through post-deployment debriefings are vital for team wellbeing and operational efficiency.

To strengthen the effectiveness, sustainability, and coordination of Ethiopia's EMTs, the following targeted measures (Box 1) should be implemented to standardize operations, enhance training, optimize resource management, and improve deployment efficiency. These strategies will also serve as a scalable model for other countries developing their N-EMTs.

Box 1 **Recommendations and next steps.**

**1. Standardizing EMT quality and achieving accreditation**

○ *Finalize and implement SOPs:* Develop standardized protocols for recruitment, rostering, administration, material and equipment management, demobilization, IPC, and WASH, with support from twinning partners.

○ *Establish an exit strategy:* Implement a commitment letter system to ensure EMT teams return equipment, maintaining long-term sustainability.

○ *Achieve WHO accreditation:* Align EMT operations with global standards to obtain WHO EMT accreditation, enhancing international recognition.

**2. Digitalizing training and capacity building**

○ *Launch an e-learning platform:* Introduce cost-effective online training for mandatory courses that do not require simulations.

○ *Pilot B-SAFE security training:* Test the platform with B-SAFE security modules, expanding to topics like conflict response and disaster risk reduction.

○ *Enhance training with case-based learning:* Develop scenario-based training addressing region-specific challenges to improve field applicability.

○ *Automate certification:* Issue digital certificates to track compliance and professional development.

○ *Strengthen multi-sectoral collaboration:* Partner with universities, military, and federal police to build a multi-agency emergency response framework.

○ *Conduct regular simulation exercises:* Implement regional field drills to enhance coordination among hospitals, health bureaus, and emergency responders.

**3. Improving data management and rostering**

○ *Upgrade the EMT rostering system:* Implement a trackable, digital roster based on international models (e.g., Poland's system) for efficient deployment tracking and data security.

○ *Ensure secure data storage:* Use encrypted cloud-based systems to protect sensitive personnel records.

○ *Automate deployment notifications:* Integrate real-time alerts to notify EMT members of deployments, urgent calls, and training updates.

**4. Strengthening logistics and resource management**

○ *Enhance field operations with mobile infrastructure:* Procure two lightweight generators and two 10,000-liter water bladders with filtration systems within 3 months.

○ *Optimize supply chain coordination:* Establish an automated inventory system to track medical supplies, equipment, and stock levels across national and regional levels.

○ *Build emergency stockpiles:* Establish regional supply depots to enable rapid response during crises.

**5. Structuring deployment and strengthening advocacy**

○ *Improve deployment coordination:* Develop a structured, transparent deployment mechanism integrating pre-deployment planning, real-time monitoring, and post-deployment evaluation.

○ *Engage stakeholders for policy alignment:* Organize regional workshops to strengthen partnerships between EMTs and health bureaus, hospitals, pharmaceutical supply agencies, and regulators.

○ *Enhance communication between national and regional teams:* Establish a dedicated EMT communication platform (e.g., secure app or dashboard) for real-time updates, resource requests, and field reporting.

**6. Enhancing psychosocial support and post-deployment wellbeing**

○ *Institutionalize mental health support:* Provide structured psychological assistance, including peer support programs, trained psychologists, and stress management training for EMT personnel.

○ *Mandate post-deployment debriefing:* Ensure all EMT members undergo structured debriefing immediately following deployments.

○ *Recognize and certify EMT contributions:* Issue certifications and official recognition to EMT personnel to acknowledge their contributions and support career advancement.

## 6 Strengths and limitations

This study is the first in the WHO Africa Region to systematically track the formation, progress, response experience, and opportunities of a fully established N-EMT. As such, it provides critical insights and a replicable framework that can guide the establishment and implementation of N-EMTs in other countries currently developing their national emergency response capacities. The study's comprehensive documentation of Ethiopia's experience offers valuable lessons for policymakers, emergency planners, and health authorities looking to standardize and strengthen EMT deployment at the national level.

However, the study also has methodological limitations inherent to a single-case study design. The primary limitation is limited generalizability, as findings from Ethiopia may not directly apply to countries with different healthcare infrastructures, governance structures, or disaster response frameworks. While the study provides a detailed, context-specific analysis, its conclusions should be interpreted with caution when applied to other settings. To address this limitation, findings were contextualized within global EMT best practices, allowing policymakers to adapt them based on country-specific challenges and capacities.

Another limitation is the risk of subjectivity in qualitative research, particularly in data interpretation. Researcher bias could influence the analysis, especially given the reliance on KIIs and retrospective data. To minimize this risk, the study incorporated triangulation, using multiple data sources, document reviews, and stakeholder perspectives to cross-validate findings. Additionally, a systematic thematic analysis framework was applied, ensuring structured and unbiased data interpretation. By corroborating information across different sources (e.g., WHO EMT network reports, government records, and firsthand accounts from EMT personnel), the study enhances its internal validity and reliability.

Despite these limitations, the study's robust methodology, use of mixed-methods research, and rigorous data validation techniques strengthen the reliability of the findings. It serves as a foundational study that not only informs Ethiopia's future EMT developments but also provides evidence-based guidance for other countries in the WHO Africa Region embarking on similar initiatives.

## 7 Conclusion

This study provides a comprehensive analysis of Ethiopia's progress in developing its emergency response mechanisms and establishing a fully functional N-EMT. It details the strategic steps taken to align the EMT system with regional and global classification guidelines, ensuring that Ethiopia's emergency response meets international standards. By examining the evolution of the EMT framework, the study highlights key lessons learned, offering a structured model that can guide other countries in developing their own N-EMTs.

The findings demonstrate that while Ethiopia has made significant strides, additional efforts are required to achieve full compliance with WHO EMT classification standards. Challenges remain in areas such as resource sustainability, operational efficiency, and workforce development, which must be addressed to enhance long-term EMT functionality. The study's targeted recommendations focus on strengthening deployment mechanisms, standardizing training, improving logistics and resource management, and ensuring financial sustainability.

Furthermore, the study underscores the critical role of continuous political commitment, cross-sectoral collaboration, and strategic investment in sustaining the N-EMT. Integrating EMTs within Ethiopia's broader health system is essential for improving coordination, streamlining communication, and ensuring long-term emergency preparedness and response capabilities. By implementing these recommendations, Ethiopia can solidify its position as a leader in N-EMT development within the WHO Africa Region, setting a precedent for other nations seeking to establish resilient and effective emergency medical teams.

## Data Availability

The datasets presented in this study can be found in online repositories. The names of the repository/repositories and accession number(s) can be found in the article/supplementary material.
